# Surgical management of a large mycotic aneurysm of the distal popliteal artery

**DOI:** 10.1016/j.jvscit.2022.07.023

**Published:** 2022-08-18

**Authors:** Kyle McCullough, Leigh Ann O’Banion, Sammy Siada

**Affiliations:** UCSF Fresno Department of Surgery, Fresno, CA

## Demographics

A 65-year-old male smoker with hypertension and coronary artery disease presents with a rapidly expanding mycotic aneurysm of the below-knee popliteal artery.

## History

The patient presented with right calf pain, swelling, and fevers after a percutaneous coronary intervention for unstable angina 2 weeks prior via ipsilateral groin access. Physical examination revealed a diffusely swollen, painful lower extremity. Ankle brachial indices were normal. Blood cultures grew methicillin-sensitive *Staphylococcus aureus*, and ultrasound examination demonstrated a sub-centimeter distal popliteal artery aneurysm with inflammation and calf deep vein thromboses. Transesophageal echocardiography and sepsis workup revealed no etiologic source. He was discharged on intravenous cefazolin and apixaban with close follow-up. Because of progression of symptoms and concern for tibial nerve compression at 6 weeks, computed tomography angiogram was obtained showing rapid aneurysm growth to 8 cm. The deep vein thromboses on repeat imaging had resolved.

## Plan

The patient underwent urgent admission and diagnostic angiography revealing a below-knee popliteal artery aneurysm extending to the tibial trifurcation with three-vessel runoff to the foot. He was taken to the operating room for repair of the aneurysm from a medial approach. The superficial femoral artery was exposed for proximal control, with distal control of the anterior tibial artery via the lateral calf and the posterior tibial artery (PTA) from a separate distal medial calf incision. Below-knee popliteal exposure revealed an obliterated popliteal and tibioperoneal trunk with a large thrombosed aneurysm. The aneurysm cavity was debrided and irrigated, and a drain was placed. The distal popliteal and proximal anterior tibial artery and PTA were ligated, thereby excluding the aneurysm. A superficial femoral artery to PTA bypass was constructed with the ipsilateral reversed great saphenous vein tunneled subcutaneously with resulting palpable pedal pulses. Intraoperative cultures were negative, and he was discharged home after an uneventful course on intravenous cefazolin. His tibial nerve compression symptoms improved, but at 6 months, he had mild paresthesias in the foot.

## Discussion

Distal peripheral arterial mycotic aneurysms present a rare and clinically challenging diagnosis. However, surgical principles of aneurysm debridement and exclusion followed by revascularization with autologous vein can lead to a successful outcome.

## Consent

Written informed consent was obtained from the patient regarding publication of his case details, video footage, and images.

## Appendix

Video ▪ ▪ ▪

